# Nonspecific symptoms dominate at first contact to emergency healthcare services among cases with invasive meningococcal disease

**DOI:** 10.1186/s12875-021-01585-8

**Published:** 2021-11-30

**Authors:** Nichlas Hovmand, Helle Collatz Christensen, Lene Fogt Lundbo, Håkon Sandholdt, Gitte Kronborg, Perle Darsø, Jacob Anhøj, Stig Nikolaj Fasmer Blomberg, Asmus Thun Bisgaard, Thomas Benfield

**Affiliations:** 1 Center for Research & Disruption of Infectious Diseases (CREDID), Department of Infectious Diseases, Copenhagen University Hospital – Amager and Hvidovre, Kettegaard Alle 30, 2650 Hvidovre, Denmark; 2 Department of Clinical Medicine, Faculty of Health and Medical Sciences, University of Copenhagen, Copenhagen, Denmark; 3 Emergency Medical Services, Capital Region of Denmark, Telegrafvej 5, 2750 Ballerup, Denmark; 4 Department of Infectious Diseases, Copenhagen University Hospital – Amager and Hvidovre, Kettegaard Alle 30, 2650 Hvidovre, Denmark; 5 Center for Health, Capital Region of Denmark, Kongens Vaenge 2, 3400 Hillerød, Denmark; 6 Diagnostic Center, Copenhagen University Hospital – Rigshospitalet, Blegdamsvej 9, 2100 Copenhagen, Denmark

**Keywords:** Infection, Emergency care systems, Prehospital care, Meningitis

## Abstract

**Background:**

An early appropriate response is the cornerstone of treatment for invasive meningococcal disease. Little evidence exists on how cases with invasive meningococcal disease present at first contact to emergency medical services.

**Methods:**

Retrospective observational study of cases presenting with invasive meningococcal disease from January 1st of 2016 to December 31st of 2020 in the Capital Region of Denmark with a catchment area population of 1,800,000. A single medical emergency center provides services to the region. Data was collected from emergency medical services’ call audio files, data from the call receiver registrations, registrations from ambulance personal and electronic health record data from the hospitalization.

**Results:**

Of 1527 cases suspected of meningitis, 38 had invasive meningococcal disease and had been in contact with the emergency service. Most contacts were to the medical helpline rather than the emergency call center at initial contact to emergency medical services. All were hospitalized within 12 h. At initial contact, fever was present in 28 (74%) of 38 cases, while specific symptoms such as headache (n=12 (32%)), a rash or petechiae (n=9 (23%)) and stiffness of the neck (n=4 (11%)) varied and were infrequent. Cases younger than 18 years of age were more often male and more often presented with fever and rash/petechiae. Only 4 (11%) received prehospital antibiotic treatment.

**Conclusions:**

Cases with invasive meningococcal disease presented with fever and unspecific symptoms. Although few were acutely ill at their initial contact, all were admitted within 12 h. We suggest that all feverish cases should be systematically asked about specific symptoms and should be wary of symptom progression to optimize the early management if cases with invasive meningococcal disease.

## Background

Currently, little evidence exists about prehospital identification and management of cases with invasive meningococcal disease (IMD) and most of the current literature is centered around timing and administration of antibiotics [1]. The evidence for other prehospital interventions for IMD is scarce. Reports on symptoms have focused on specific symptoms such as fever, rashes or meningeal symptoms, while other early symptoms that have been reported in children, such as leg pain, have not been assessed in adult patients [2, 3]. A study of early symptoms in young infants with bacterial meningitis (BM) indicated that non-specific features associated with bacterial meningitis rarely progressed from onset to admission [4]. Similar studies among non-neonatal cases and cases with IMD are needed.

IMD remains a significant global burden of disease, even though effective treatment is widely available [5–8]. IMD presenting as sepsis is more lethal than when presenting as meningitis [9]. Classical features of meningococcal sepsis include a characteristic petechial rash in affected cases with fever, but the early symptoms of the disease are often nonspecific, thus making an early diagnosis difficult [10, 11]. Lack of early recognition of the disease can within a matter of hours lead to a significantly increased risk of death or permanent injury [6, 11]. 85% of deaths from IMD has been reported to occur within 24 h from diagnosis [12]. More knowledge about early symptoms of IMD is needed to increase the number of cases who receive treatment early.

In the Capital Region of Denmark contact to the emergency medical services (EMS) generally follows two paths. One contact telephone number is for absolute medical emergencies that receives 130,000 health related annual calls. The other telephone contact number is to a medical helpline staffed by a registered specialist nurse that receives 950,000 annual calls. The region’s guideline for prehospital handling of cases suspected of IMD is that an ambulance and a medical doctor should be sent to the case immediately and if IMD is suspected, antibiotics should be administered on site.

Here we report information about symptoms presenting in IMD cases at the first contact to EMS.

## Methods

### Case identification

This is a retrospective observational study of cases presenting with IMD from January 1st of 2016 to December 31st of 2020 in the Capital Region of Denmark with a catchment area population of 1,800,000. The Committee on Health Research Ethics were not involved in approving the study as this was a quality development project. Permission to collect data from case records was granted by Center for Health and by Emergency Medical Services in the Capital Region of Denmark as required by Danish legislation [13].

All Danish residents have a unique personal identification number permitting linkage to national health registries. Eligible cases were identified by assessing all diagnosis codes of meningitis (International Classification of Disease, 10th edition, (ICD-10) codes: DG00*) or IMD (ICD-10: DA39*) in electronic health records (EHR). Further, the databases of the region’s clinical microbiology services were reviewed, as patients with any positive finding in a sample of cerebrospinal fluid and all patients with a positive finding of meningococci from any anatomical location were added to the list of potential cases to be screened. Each case’s EHR was reviewed by a physician.

### Data collection

Data sources included EMS call audio files, data from the call receiver registrations, registrations from ambulance personal and EHR data from each individual hospitalization. All audio files and records were abstracted by a physician. It was noted whether a symptom was mentioned or if the symptom was present during the conversation or registered by ambulance personal.

Data on treatment initiation prior to hospitalization were gathered from ambulance registrations and/or EHRs, while data on age, gender, time to initiation of relevant treatment during hospitalization and on 30-day mortality were gathered from EHRs. Data on serogroups and sites of infection were gathered from a database containing all clinical microbiological tests in the Capital Region of Denmark.

### Statistical analysis

Values are presented as median and interquartile ranges or proportions and percentages. Correlations were calculated using Spearman’s rank correlation coefficient. *P*-values for differences between age groups were calculated using Fisher’s Exact Test. Two-sided *P* values of less than 0.05 were considered statistically significant. Statistics were done in R version 3.6.0. Because symptoms were not always asked about, *P* values are presented for both symptom present in cases asked about that symptom and symptom present in all cases. *P* values are reported as crude values not adjusted for multiple comparisons.

## Results

### Case characteristics

**Fig. 1:**
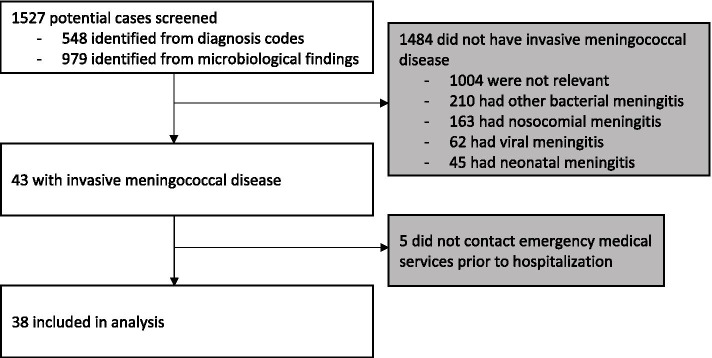
Flow chart of case identification, inclusion and exclusion. This flow chart illustrates the sources that cases were identified from and reasons for exclusion. Following this process, 38 cases with invasive meningococcal disease who had contact to emergency medical services prior to hospitalization between 2016 and 2020 in the Capital Region of Denmark were included in analysis

A total of 1527 potential cases were identified; 548 cases by ICD-10 codes and 979 identified through clinical microbiology databases. Each case’s EHR was evaluated. Forty-three cases had confirmed IMD. Five had no contact to the EMS prior to hospitalization. The remaining 38 cases were included for analysis (Fig. 1). Thirty-five cases were confirmed by culture while 3 cases were confirmed by polymerase chain reaction.

The cases were evenly distributed over the 5-year period, except for 2020 where there were fewer cases (Table 1). The median age was 20 years, and 22 (58%) were male. Twenty (53%) had sepsis, 8 (21%) had meningitis, and 10 (26%) had sepsis and meningitis. Twelve (32%) were infected with serogroup B, 5 (26%) with C, 10 (26%) with W, 8 (21%) with Y and 3 (8%) with an unknown serogroup. Serogroup B was the most common in children while serogroup W was the most common in adults (Table 1). Thirteen (34%) initially contacted the emergency call center, while the medical helpline was the first contact for 25 (66%).

**Table 1:** Case characteristics

	All N = 38	Age <18 years N = 17	Age ≥18 years N = 21	*p*-value
Age (years)				-
- Median (interquartile range)	20 (5 and 61)	3 (1 and 16)	60 (37 and 77)	
Gender				<0.01
- Female	16 (42%)	2 (12%)	14 (67%)	
- Male	22 (58%)	15 (88%)	7 (33%)	
Site of infection				
- Meningitis	8 (21%)	5 (29%)	3 (14%)	0.43
- Meningitis and sepsis	10 (26%)	5 (29%)	5 (24%)	0.72
- Sepsis	20 (53%)	7 (41%)	13 (62%)	0.32
Serogroup				
- B	12 (32%)	8 (47%)	4 (19%)	0.09
- C	5 (13%)	2 (12%)	3 (14%)	1.00
- W	10 (26%)	2 (12%)	8 (38%)	0.14
- Y	8 (21%)	3 (18%)	5 (24%)	0.71
- Unknown	3 (8%)	2 (12%)	1 (5%)	0.58
Year at disease				
- 2016	8 (21%)	2 (12%)	6 (29%)	0.26
- 2017	8 (21%)	4 (24%)	4 (19%)	0.69
- 2018	11 (29%)	8 (47%)	3 (14%)	0.02
- 2019	8 (21%)	2 (12%)	6 (29%)	0.26
- 2020	3 (8%)	1 (6%)	2 (10%)	1.00
Emergency service used				0.08
- 112: Emergency call center	13 (34%)	3 (18%)	10 (47%)	
- 1813: Medical helpline	25 (66%)	14 (82%)	11 (53%)	
30-day mortality				0.31
- Survivor	34 (89%)	14 (82%)	20 (95%)	
- Non-survivor	4 (11%)	3 (18%)	1 (5%)	

List of case characteristics of the 38 cases with invasive meningococcal disease who had contact to emergency medical services prior to hospitalization between 2016 and 2020 in the Capital Region of Denmark. Cases are grouped as either children under 18 years of age or adults. Children were more likely to be male while adults were more likely to be female. In 2018 there were more children compared to adults. No other differences between the age groups were significant

Cases younger than 18 years of age were more often male, while those older than 18 were more often female (Table 1, p<0,01). In 2018, more cases were younger than 18 years of age than older (p=0.02). There were no other significant differences between the age groups regarding site of infection, serogroup, year of disease, emergency service used or 30-day mortality.

### Symptoms at initial contact to EMS

At least one symptom was mentioned in each call but in no call all symptoms were mentioned (Table 2). The most common symptom was fever that was present in 28 of the 30 cases that were asked about or mentioned it during the call followed by fatigue (20 of 23). Specific symptoms associated with IMD or meningitis were not as often confirmed to be present during the initial contact: headache (12 of 14), altered mental state (10 of 26), leg pain (9 of 10), rash or petechiae (9 of 18), stiffness of the neck (4 of 12) and photophobia (1 of 1). Children and adolescents were significantly more likely to present with fever and rash/petechiae compared to adults who often a difficulty breathing.

**Table 2:** Symptoms at initial call to emergency medical services

	Symptom present / symptom asked about			*p*-value	
	All cases N = 38	Age <18 years N = 17	Age ≥18 years N = 21	present of asked about	present of all cases
Fever	28 of 30	16 of 17	12 of 13	1.00	0.01
Fatigue	20 of 23	11 of 14	9 of 9	0.25	0.21
Headache	12 of 14	6 of 8	6 of 6	0.47	0.73
Vomiting	12 of 18	6 of 9	6 of 9	1.00	0.73
Upper airway symptoms	10 of 16	5 of 8	5 of 8	1.00	0.73
Difficulty breathing	10 of 19	1 of 5	9 of 14	0.14	0.01
Altered mental state	10 of 26	5 of 13	5 of 13	1.00	0.73
Leg pain	9 of 10	4 of 5	5 of 5	1.00	1.00
Rash and/or petechiae	9 of 18	9 of 15	0 of 3	0.21	<0.01
Tremors and/or seizures	7 of 7	2 of 2	5 of 5	1.00	0.43
Diarrhea	6 of 7	2 of 2	4 of 5	1.00	0.67
Stiffness of the neck	4 of 12	3 of 9	1 of 3	1.00	0.31
Chest pain	3 of 6	0 of 0	3 of 6	1.00	0.24
Abdominal pain	3 of 6	0 of 1	3 of 5	1.00	0.24
Sparse urination	2 of 6	0 of 3	2 of 3	0.40	0.49
Photophobia	1 of 1	1 of 1	0 of 0	1.00	0.45
Endangered airway	1 of 4	0 of 1	1 of 3	1.00	1.00
Stroke-symptoms	1 of 4	0 of 0	1 of 4	1.00	1.00

List of symptoms mentioned in 38 initial phone calls to emergency medical services. Thirteen calls were to the emergency call center 112, while 25 were to the medical helpline 1813. For any symptom, it was registered in how many calls the symptom was present and in how many calls the symptom was asked about and/or mentioned. Because symptoms were not always asked about, *p-*values are presented for both symptom present in cases asked about that symptom and symptom present in all cases. There was no difference between children and adults in symptoms present of symptoms asked about, but of all cases, children more often presented with fever and rash/petechiae than adults

**Fig. 2:**
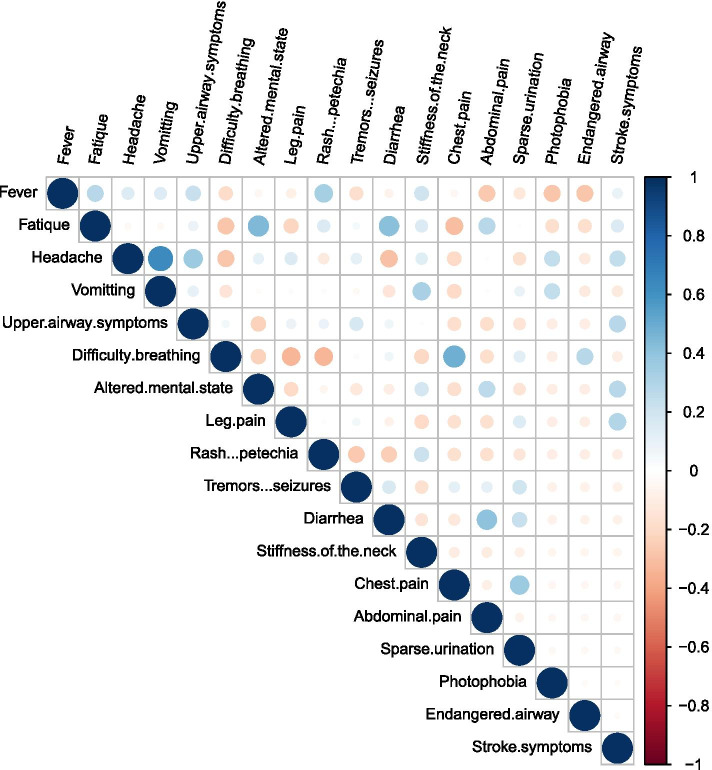
Paired correlations between symptoms. Plot showing paired correlations between symptoms at first contact to emergency medical services for the 38 cases with invasive meningococcal disease who had contact to emergency medical services prior to hospitalization between 2016 and 2020 in the Capital Region of Denmark. The highest correlations were seen between headache and vomiting (r=0.63, CI95%: [0.40;0.79], p<0.01), difficulty breathing and chest pain (r=0.49, CI95%: [0.20;0.70], p=0.27) and fatigue and altered mental state (r=0.45, CI95%: [0.14;0.67], p=0.73)

Of the 18 cases asked about or mentioning both fever and a rash or petechiae, 9 cases presented both. Of the 8 cases asked about or mentioning both fever, headache, and stiffness of the neck, 2 cases presented all three symptoms. The highest paired correlations were seen between headache and vomiting (r=0.63, CI95%: [0.40;0.79], p<0.01), difficulty breathing and chest pain (r=0.49, CI95%: [0.20;0.70], p=0.27) and fatigue and altered mental state (r=0.45, CI95%: [0.14;0.67], p=0.73) (Fig. 2).

### Prehospital management

**Fig. 3:**
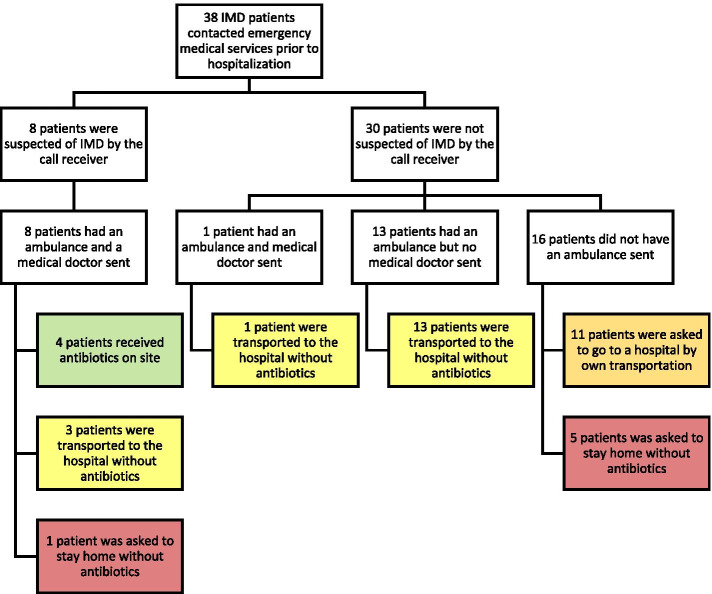
Flow chart of prehospital management of cases with invasive meningococcal disease. Thirty-eight cases who had invasive meningococcal disease (IMD) called emergency medical services prior to hospitalization. This flow chart illustrates how cases were handled prehospitally. Four of the 38 cases received antibiotics on site after evaluation from a medical doctor as per the region’s guideline (green). Seventeen cases were transported to a hospital by an ambulance but did not receive treatment (yellow). Eleven cases were asked to go to the hospital by own transportation (orange). Six cases were asked to stay home (red). All 38 cases were hospitalized by one way or another within 12 h

From the 38 initial phone calls, 8 cases were suspected of IMD by the call receiver (Fig. 3). All 8 were seen prehospitally by a medical doctor and 4 of the cases received antibiotics on site. Of the remaining 4 cases, 3 were taken to the hospital while 1 was asked to stay at home.

None of the 30 cases not suspected of IMD during the phone call received prehospital antibiotics. A single case was seen prehospitally by a medical doctor and was then taken to the hospital. Ambulances were sent to 13 of the 30 cases and they were all taken to the hospital. Of the remaining 16 cases that were not seen prehospitally, 11 were asked to transport themselves to the hospital while 5 were asked to stay at home after the initial phone call. Those 5 cases all were hospitalized within 6 h, as one went to the hospital by own transportation, one called the medical helpline again and three called the emergency call center.

All 38 cases were hospitalized one way or another within 12 h from the initial phone call.

### Hospital management and outcome

**Fig. 4:**
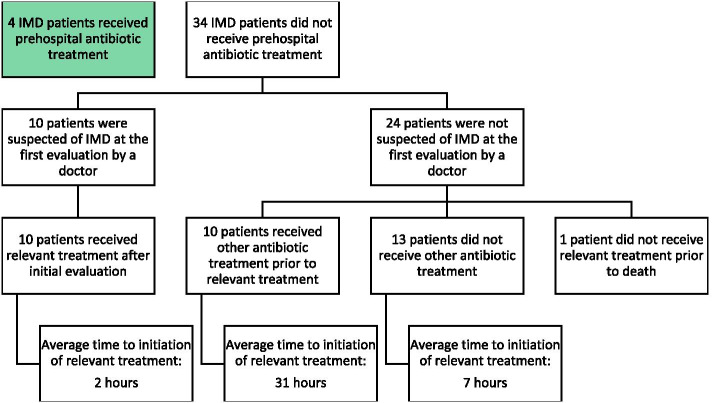
Flow chart of hospital management of cases with invasive meningococcal disease. Thirty-four cases who had invasive meningococcal disease (IMD) arrived at a hospital without being treated prehospitally. Ten were suspected of IMD at first evaluation and treated as such. Twenty-four cases were not suspected of IMD at first evaluation. Ten of these initially received other antibiotic treatment while 13 did not receive other antibiotic treatment before initiation of relevant treatment. One case did not receive relevant treatment prior to death. Relevant treatment was defined as treatment against a known etiology or empirical treatment as per local guideline for suspected bacterial meningitis or invasive meningococcal disease

Of the 34 cases that arrived at the hospital without having received antibiotics prehospitally, 10 were suspected of IMD at the first evaluation and treated as such with an average time to initiation of relevant treatment of 2 h (Fig. 4). Relevant treatment was defined as treatment against a known etiology or empirical treatment as per local guideline for suspected IMD or BM.

Thus, 24 cases were not suspected of IMD at first evaluation at the hospital and 10 of these initially received other antibiotic treatment before they received relevant treatment after an average of 31 h. A single case in this group did not survive. Thirteen cases not suspected of IMD did not receive other antibiotic treatment before initiation of relevant treatment after an average of 4,5 h. There were 2 cases in this group that did not survive, while 1 case died before receiving any antibiotic treatment.

Thirty-day mortality was 11% (4/38) and all fatalities occurred within the first 24 h from initial contact.

## Discussion

Early suspicion and identification of cases with IMD was difficult because symptoms were diverse and nonspecific at the initial contact to the EMS. The most common symptom was fever, while specific symptoms associated with IMD were less frequenty confirmed to be present and were rarely mentioned or asked about during the initial contact to the EMS. Symptoms differed by age suggesting that age-guided questioning may be relevant. While some specific symptoms have a high specificity for severe infections, patients presenting with nonspecific symptoms can have a noteworthy risk of short-term mortality across a broad spectrum of conditions [14, 15]. While all feverish cases should be asked about specific symptoms, such as rashes, headache, stiffness of the neck and strong leg pain, cases younger than 18 years of age with fever and a rash require additional attention and should be cautioned of symptoms and signs suggestive of progression and general worsening of their condition. In contrast, older individuals more often reported difficulty breathing. We acknowledge that fever and rash in children and adolescents occur frequently due to several mainly viral etiologies. Similarly, difficulty of breathing is a frequent symptom among adults.

Larger cohorts and further research are warranted to identify patterns of early presenting symptoms that may better discriminate individuals with a high likelihood of IMD. Current literature on this subject is very limited, especially among adults. Our findings elaborate the current knowledge about how nonspecific symptoms dominate at early stages of IMD, but our data also indicate that cases with IMD progress rapidly to life-threatening disease, as all cases were hospitalized within 12 h, regardless of which symptoms they presented with. When assessing a febrile child, general practitioners rarely consider BM or IMD as likely causes and prehospital penicillin is mainly given if the diagnosis of meningitis or septicaemia is thought to be certain [16]. Conflicting data have been reported about the effects of prehospital antibiotic treatment which may be because the antibiotics more often were given to patients with more severe IMD and thus higher risk of bad outcome [17]. However, more recent studies have found that prehospital antibiotics can be protective against death from IMD and that it is a safe treatment, even though more data is needed to either recommend or reject the use of it [18, 19]. We believe that relevant treatment should be initiated as soon as possible when suspecting a time critical condition such as IMD.

We suggest that all healthcare workers, such as general practitioners and EMS-workers, who may have early contact to feverish patients consider IMD even when specific symptoms are absent. It is important to keep in mind that not all cases of IMD have BM, thus ruling out BM does not rule out IMD. In addition, we suggest that feverish patients who is not suspected for IMD are informed about specific symptoms and are encouraged to contact medical services again if their symptoms progress, especially within the first 12 h from the initial contact. A study has shown that in children with IMD parents often found their children’s behavior and the course of the disease to be different from previous illnesses [20], thus it is important to include their perception when considering diagnoses in febrile children.

The main limitations of this study are the modest sample size and the fact that symptoms may have been present during the initial contact to emergency medical services even if they were not asked about. This limits our ability to perform statistical analyses, but we do believe that the general picture of symptoms in this study is representable for how cases with IMD present at first contact to EMS. The structure of acute healthcare systems varies across the world, thus cases with IMD may present differently in other countries. We believe that we were able to include most cases with IMD in our analysis because we used very broad criteria for screening as indicated by the many excluded cases. We may have missed cases due to prehospital deaths from IMD. However, all forensic autopsies are performed on unexpected deaths in Denmark, which may have led to findings of meningococci. Thus, we find it unlikely that cases were missed.

We present novel unique information of all stages of IMD from initial EMS calls to outcome. This method could be introduced for larger populations where similar data is available and for other diseases where the initial handling of the disease is vital for outcome, such as BM.

## Data Availability

All data generated or analyzed during this study are included in this published article.

## Abbreviations

BM: Bacterial meningitis
EHR: Electronic health record
EMS: Emergency medical services
IMD: Invasive meningococcal disease
